# Treating White Spot Lesions and Non-Carious Cervical Lesions with Amelogenin Peptide-Based Hydrogel

**DOI:** 10.3390/biomimetics10020120

**Published:** 2025-02-18

**Authors:** Erika Bauza Nowotny, Salony Jassar, Jin-Ho Phark, Janet Moradian-Oldak

**Affiliations:** 1 Center for Craniofacial Molecular Biology, Herman Ostrow School of Dentistry, University of Southern California, Los Angeles, CA 90089, USA; nowotny@usc.edu (E.B.N.); sjassar@usc.edu (S.J.); 2 Department of Operative Dentistry, Herman Ostrow School of Dentistry, University of Southern California, Los Angeles, CA 90089, USA; phark@usc.edu; 3 Department of Biomedical Engineering, Viterbi School of Engineering, University of Southern California, Los Angeles, CA 90089, USA

**Keywords:** biomimetic, peptide, hydrogel, white spot lesions, non-carious cervical lesions

## Abstract

Peptide-based biomimetic treatments have gained increased attention in the dental field due to their biocompatibility and minimally invasive qualities. These biomimetic approaches can replicate the native architecture of dental tissues, thus contributing to higher success rates and improved longevity of restorations. The aim of this study was first to examine the biocompatibility and stability of an amelogenin peptide-based chitosan hydrogel (P26-CS) against salivary enzymes. Second, we aimed to evaluate its efficacy in biomimetically repairing human dental lesions in situ. White spot lesions (WSLs) in enamel and non-carious cervical lesions (NCCLs) in dentin were artificially created. Chitosan (CS) improved peptide stability, while remineralization of enamel sections with P26-CS was not impeded by salivary enzymes. The peptide was not cytotoxic, irritating, or sensitizing. Fluorescently labeled P26-CS penetrated ~300 μm into the enamel of WSLs and ~100 μm into the dentin of NCCLs. After peptide treatment, quantitative light-induced fluorescence (QLF) and microcomputed tomography (μCT) indicated a gain in mineral density of WSLs. In NCCLs, scanning electron microscopy showed that the dentin was covered by a mineral layer of needle-shaped crystals. Our results show that the repair of artificial WSLs and NCCLs was achieved by P26 peptide-guided remineralization and demonstrate its potential to repair dental lesions.

## 1. Introduction

The management of dental lesions, particularly white spot lesions (WSLs) and non-carious cervical lesions (NCCLs), poses a significant challenge in clinical dentistry. Traditional restorative techniques often fall short in effectively addressing these conditions due to their inability to replicate the native structure of enamel and dentin, leading to sub-optimal retention rates and compromised esthetic outcomes [1,2,3]. Non-invasive treatments have been proposed for the prevention and treatment of WSLs. For example, casein phosphopeptide-stabilized amorphous calcium phosphate (CPP-ACP) was shown to prevent enamel demineralization and further promote the regression of coronal caries [4]. However, when tested in randomized controlled or double-blinded studies, the effectiveness of CPP-ACP could not be conclusively verified and often scored equally to fluoride varnish [5,6]. Better enamel remineralization has been observed with self-assembling peptides [7]; however, details of the microstructure of the regrown enamel have not been investigated.

NCCLs present unique challenges for successful restoration [8], as common restorative materials, namely resin composites and glass ionomers, have poor retention rates [9,10]. Therefore, it is vital to develop a new treatment option that can biomimetically remineralize the affected dentin and enamel. This would not only decrease the hypersensitivity associated with exposed dentin, but potentially also provide a remineralized dentin substrate upon which traditional restorative materials can bind. Overall, current treatments fail to effectively mimic or replicate the native enamel and dentin structure. Since structure is intimately tied to function, the longevity of the restorations and resistance to daily activity (stress, acidic challenges, etc.) is a cause for concern. Thus, it is imperative to develop new biomimetic treatments for WSLs and NCCLs that focus on replicating the structure and mechanical properties of healthy enamel while aiming to maximize the preservation of natural tooth structure.

In recent years, there has been a growing interest in peptide-based biomimetic treatment modalities as innovative alternatives that not only enhance biocompatibility but also offer minimally invasive solutions for dental repair [11,12,13,14]. Among these, amelogenin peptides have emerged as promising agents capable of promoting remineralization and structural restoration of damaged dental tissues [15,16,17]. Amelogenin is the main protein component in the enamel extracellular matrix, playing crucial role in crystal growth and organization during enamel formation [18].

In our laboratory, we have utilized P26, a rationally designed 26-amino-acid amelogenin peptide that preserves key sequences from the full-length amelogenin protein. More specifically, P26 contains apatite-binding, self-assembly, and mineralization-promoting domains. P26 has been shown to separately rebuild enamel, remineralize dentin, and establish a strong material–tooth interface in in situ models. More specifically, P26 has been shown to enhance the nucleation of small, uniformly sized apatite crystals and promote the development of multiple organized aprismatic enamel-like layers on etched enamel [11]. In dentin, P26 was shown to promote the mineralization of collagen fibrils [19].

A wide body of research has documented the use of chitosan (CS) in medicine. This polymer has been used as a main component of gels for the treatment of joint pain, tissue engineering, and even neurogenerative diseases [20]. Chitosan is an exceptional vehicle for drug delivery in tissue engineering, as it possesses a myriad of properties such as non-toxicity, biodegradability, bacteriostaticity, and mechanical and thermal stability [21,22,23].

Additionally, studies have investigated the use of CS in dentistry for a variety of treatments, including periodontitis and remineralization [20,24]. While shown to enhance enamel remineralization by itself [25], CS has been more often used as a carrier for proteins [26], nanoparticles [27], and a variety of complexes (i.e., peptide–ACP) [28] while treating/repairing demineralized enamel lesions. We therefore used CS as the carrier of our peptide, creating a CS peptide-based hydrogel.

Our previous systematic investigation of amelogenin–chitosan interactions under different pH conditions revealed that their interactions were pH-dependent [29]. More specifically, at pH values below 6.5 (the pKa of chitosan), the increase in charge density facilitated strong electrostatic interactions between chitosan and amelogenin. At pH values above 6.5, the interaction was significantly weaker due to the decreased solubility of chitosan and deprotonation of its amino groups. Thus, above pH of 6.5, amelogenin did not interact with CS and was the dominant factor governing the oriented growth of fluoridated hydroxyapatite crystals. It was therefore suggested that at neutral pH, the interactions between amelogenin and CS were not significant and amelogenin could be available to regulate enamel remineralization. Additionally, incorporation of our peptide P26 in CS conferred extended peptide stability at room temperature and at 37 °C [13].

We then examined the efficacy of our peptide–chitosan hydrogel in remineralizing enamel and dentin [12,13]. Additionally, P26-CS facilitated the regeneration of enamel-like hydroxyapatite crystals, providing substantial enhancements to both the mechanical characteristics (hardness and elastic modulus) and the mineral density of demineralized enamel lesions [13]. Furthermore, P26-CS successfully promoted biomimetic remineralization of the dentinal collagen matrix, by dentinal tubule occlusion and rebuilding a protective mineralized layer on the demineralized dentin surface [12].

Here, we utilized an amelogenin-derived peptide–CS hydrogel (P26-CS) to assess its efficacy in repairing WSLs and NCCLs. We first analyzed the stability of the hydrogel against salivary enzymes in solution. We then used a model of demineralized enamel sections to evaluate the efficacy of P26-CS hydrogel in remineralizing demineralized enamel. The null hypothesis assumed equal mineral density of enamel sections with or without P26-CS treatment, in the presence or absence of salivary enzymes. Using confocal microscopy to visualize a fluorescently labeled P26 peptide, we examined the penetration depth of the peptide into WSLs and NCCLs during the remineralization process. The gain in mineral density following treatment of WSLs with P26-CS was measured by Quantitative Light-Induced Fluorescence (QLF) and μCT. The null hypothesis assumed equal mineral density of enamel blocks with or without treatment and that the observed distribution of % bone mineral density recovery across control and treated groups matched the expected distribution (i.e., no significant difference). Finally, the potential of P26-CS in repairing NCCLs was investigated using an artificial NCCL model on premolars.

## 2. Materials and Methods

### 2.1. P26 Peptide Synthesis and Characterization of Degradation Products Without Enzymes

P26 amelogenin peptide was synthetized by Biomer Technology (San Francisco, CA, USA). P26 solutions (0.2 mg/mL) were prepared as previously described [11,29] and incubated at 37 °C for 7 days and subsequently analyzed by liquid chromatography/mass spectrometry (LC/MS; Scripps Research Institute, San Diego, CA, USA). Significant peaks in the MS spectra were matched to degradation products of P26 based on their m/z.

### 2.2. Enzymatic Digestion of P26 by Salivary Enzymes

Solutions of MMP8, MMP1 (R&D systems, Minneapolis, MN, USA), α-amylase, and lysozyme (Sigma, Burlington, MA, USA) were prepared following the manufacturer’s instructions. Prior to each use, pro-MMP1 and pro-MMP8 were activated with 10 mM APMA according to the manufacturer’s protocol (R&D Systems). Successful cleavage of both enzymes into their active form was confirmed using SDS-PAGE gel electrophoresis. To better mimic in vivo oral conditions, all enzymes were used at concentrations found in healthy individuals, as reported in the literature: MMP8 (final 0.286 μg/mL; [30], MMP1 (final 20 pg/mL [31], α-amylase (final 119 U/mL; [32], and lysozyme (final 0.4 μg/mL [30]. A total of 125 μL of aqueous P26 or P26-CS solutions (0.2 mg/mL final) was added to an equal volume of artificial saliva (AS; 1.2 mM CaCl_2_·2H_2_O, 50 mM HEPES buffer, 0.72 mM KH_2_PO_4_, 16 mM KCl, 4.5 mM NH4Cl, 0.2 mM MgCl_2_·6H20, and 1 ppm NaF). Then, the enzymes were mixed in and immediately placed in a water bath at 37 °C. An initial collection was (t = 0) carried out and analyzed by Reverse-phase High-Performance Liquid Chromatography (HPLC), as described below. Incubations for each sample were performed in triplicate. To quantify the effect of each enzyme, individual enzymes were incubated with 125 μL of P26-CS and 125 μL of AS. The enzymes that had the highest (α-amylase) and lowest concentrations (MMP1) were also tested in two additional concentrations: α-amylase (final 23.87 U/mL; 1:5 dilution) and MMP1 (final 2 ng/mL; 1000×).

#### HPLC Analysis

At given time points (30 min, 5 h, and 24 h), the samples were removed from the water bath, lightly vortexed, and 75 μL of each sample was mixed with 125 μL 0.1% trifluoroacetic acid (TFA) prior to HPLC injection. HPLC analysis was performed following each collection using a reverse-phase C18 column (Phenomenex, Torrance, CA, USA). Peptide peaks were analytically quantified, using peak area as a measure of peptide stability.

### 2.3. Biocompatibility Evaluation

Good laboratory practice (GLP) biocompatibility evaluation was performed to test the P26-CS hydrogel for in vitro cytotoxicity, dermal sensitization, and oral mucosa irritation. This evaluation was performed by NAMSA (Northwood, OH, USA) in compliance with the International Organization for Standardization (ISO) 10993-5 and 10993-10 and consistent with FDA guidance for the evaluation of medical devices.

### 2.4. Remineralization of Enamel Sections with Salivary Enzymes and Their Analysis

Extracted human molars were obtained as coded specimens following standard procedures at the Herman Ostrow School of Dentistry of USC. There was no direct identification of the patients from which teeth samples were collected. The selected specimens were free of cracks, caries, or restorations. The molars were cleaned and sectioned longitudinally into ~1.5 mm sections using a water-cooled low-speed diamond saw (SYJ-160, MTI Corporation, Richmond, CA, USA). Then, the sections were immersed in DI water and cleaned in an ultrasonic bath (Branson 2510 Ultrasonic Cleaner, Bransonic, Danbury, CT, USA) for 5 min twice. The molars were stored in a phosphate-buffered saline (PBS, pH 7.4) solution with 0.002% (*w*/*v*) sodium azide at 4 °C until ready to use. The sections were painted with clear nail varnish, leaving two 1 × 1 mm^2^ windows exposed in enamel. Samples were dried for 2 h before treatment application. Enamel sections were demineralized in 20 mL of demineralization solution (2 mM CaCl_2_·2H_2_O, 2 mM KH_2_PO_4_, 50 mM sodium acetate, and 0.05 M acetic acid) at pH 4.6 for 5 days at 37 °C as previously described [13]. The sections randomly distributed into one of four groups (1) control + enzymes, (2) P26-CS + enzymes, (3) control no enzymes, or (4) P26-CS no enzymes. After demineralization, samples were rinsed, sonicated, and placed in sterile vials containing 3 mL of artificial saliva at 37 °C for 5 days. P26-CS (0.2 mg/mL) was applied to one of the two windows on days 0, 3, and 5. Artificial saliva solution was changed every day, while activated enzymes were added to artificial saliva every other day. The sections were then examined by QLF as described below.

#### Quantitative Light-Induced Fluorescence (QLF)

To determine the change in mineral density of the lesions on enamel sections, QLF images were taken at baseline, post-demineralization, and post-remineralization time points. QLF-D Bioluminator 2 camera settings (Inspektor Research System, Bussum, The Netherlands) were as follows: (ISO 1600, aperture 18, shutter speed 1/125 s) and under blue light (ISO 1600, aperture 5.6, shutter speed 1/30 s). To ensure consistency in imaging at the different time points, each specimen was placed in a custom-made mold. This system ensured consistent positioning of the sample for different measurements as well as standardization of the distance between specimen surface and the lens. In the blue light image, a region of interest was drawn circumscribing the lesion and surrounding healthy enamel and the ΔF (i.e., loss of fluorescence) was recorded. The ΔF value of each lesion was computed as the average of three measurements. The final ΔΔF value was calculated as follows:

(1) $$∆F\;demin=F\left(Demin\right)-F\left(Baseline\right)$$

(2) $$∆∆F=∆F\left(Remin\right)-∆F\left(Demin\right)$$

Positive ΔΔF values indicate a net mineral gain, while negative ΔΔF indicates mineral loss [13]. Statistical analysis for enamel sections included One-Way ANOVA, conducted with GraphPad Prism 9.1 (San Diego, CA, USA).

### 2.5. Penetration Experiments of Peptides into In Situ WSLs and NCCLs During Remineralization

#### 2.5.1. Preparation of Artificial NCCLs

Healthy human premolars were cleaned and sonicated. Prior to brushing, a 3D scan (intraoral scanner Medit i500, Seoul, Republic of Korea) and white-light pictures (Leica MDG41, Wetzlar, Germany) were obtained from each specimen. The premolars were assembled in custom molds in pairs of similar size and aligned at the cemento-enamel junction (CEJ). With the buccal side facing up, polymethyl methacrylate (PMMA, Jet Liquid, Wheeling, IL, USA) was poured into the mold to embed the teeth with the buccal side exposed. A festooned artificial gingiva using a periodontal dressing (Barricaid, Dentsply, Milford DE, USA) was created [10]. A toothpaste slurry (3:1 ratio of toothpaste to water) was prepared using fluoride free, medium abrasiveness toothpaste (Tom’s of Maine Whitening toothpaste, Kennebunk, ME, USA). The slurry was poured into each mold to cover the teeth. NCCLs were created by horizontal brushing with a dual axis chewing simulator (CS-4.8, SD-Mechatronik, Feldkirchen-Westerham, Germany). A soft toothbrush with parallel bristles (Crest, Oral B Indicator, flat trim, 35 soft, Cincinnati, OH, USA) was attached to each simulator mechanical arm, which brushed the samples at 45 mm/s for 70,000 cycles. After the brushing, samples were rinsed with DI water, sonicated for 5 min, and 3D scans and white-light pictures were obtained.

#### 2.5.2. Preparation of Blocks (WSLs)

Healthy molars were sectioned into 4 blocks, cleaned and sonicated as described previously. Clear nail varnish was applied to the block leaving one 2 × 2 mm^2^ window exposed in the enamel. Samples were dried for 2.5 h before application of P26-CS for remineralization treatment. To create in situ white spot lesions, the blocks were demineralized for 15 days [12]. Prior to treatment, the samples were rinsed and sonicated for 5 min. The blocks were randomly assigned to a treatment group: negative control (untreated), P26-CS (0.5 mg/mL), and fluoride varnish (Sparkle V 5% Sodium Fluoride Varnish with Xylitol, Des Plaines, IL, USA).

#### 2.5.3. Peptide Penetration into WSLs and NCCLs

P26 peptide was custom labeled with Cy5 dye via a cysteine conjugation (Chempeptide, Shanghai, China) to enable imaging through confocal microscopy. After etching, the WSLs were treated with Cy5 P26-CS hydrogel containing 0.5 mg/mL peptide (P26:Cy5-Cys-P26; 1:2) for 30 min. Control groups included: CS only, etching only, and healthy enamel (no WSL). When assessing the penetration in the presence and absence of enzymes, the blocks were treated with Cy5 Cys-P26 subsequently incubated in 3 mL of AS for 2 h at 37 °C prior to confocal analysis. Activation of enzymes and concentrations were as described previously. An in situ NCCL and a natural NCCL were etched, treated with Cy5 Cys-P26 and remineralized for 1.5 h in AS at 37 °C.

#### 2.5.4. Confocal Microscopy

WSL and NCCL specimens were analyzed by laser scanning confocal microscopy (Leica Stellaris 5; objective: HCPL FLUORAR 5×). The WSL blocks were placed in a glass bottom EZ-slide with 1 mL of DI water and analyzed. The penetration depth of the peptide was assessed through a combination of z-stack images (recorded using an optical pitch of 2 μm) which were analyzed using LAS-X software (version 4.5.0.25531). Cy5-P26 was detected at 493 to 643 nm (excitation 488 nm) and dentin autofluorescence at 643 to 817 nm (excitation 638 nm). Samples were imaged again after a 7-day remineralization in AS at 37 °C.

For NCCL specimens, 3D volumetric reconstructions were used which were then optically segmented at maximum-intensity projections to evaluate peptide penetration along the XZ plane. Penetration along the XZ plane was calculated by averaging 2–3 measurements independently extracted from 2 to 3 different areas within the lesion. The in situ NCCL was remineralized and analyzed again after 7 days.

### 2.6. Remineralization of Enamel in White Spot Lesions (WSL)

Specimens in the P26-CS group were etched with 37% phosphoric acid for 20 s and rinsed with DI water. In a custom mold lined with parafilm, an indent was made around the lesion area which created a pouch that would hold the hydrogel. 40 μL of P26-CS was applied to the pouch, and the block was placed with the lesion facing down on the pouch. Finally, the parafilm was wrapped around the block. During the treatment (30 min or 6 h), the block was removed from the mold and placed with the lesion facing up on the bench. Fluoride varnish (FV) was used as a positive control and applied following manufacturer’s instructions. Following FV application, the sample was remineralized in artificial saliva for 3 h. FV was subsequently removed from the enamel surface by immersion in a small amount of chloroform for 10 s. Following treatment, all samples were immersed in autoclaved glass vials containing 5 mL of artificial saliva at 37 °C for 21 days. The AS solution was replaced every day and the vials replaced every other day. P26-CS re-applications were performed 2×/week in the 30 min group and 1×/week in the 6 h group. After remineralization was complete, the samples were sonicated for 5 min and rinsed. The gain in mineral density was analyzed by QLF as described above and Microcomputed Tomography (μCT) as described below. Statistical testing of blocks QLF data included One-Way ANOVA. In the experiment that included QLF + μCT data, tests for homogeneity of variance indicated unequal standard deviations, suggesting that a Brown–Forsythe and Welch ANOVA would be appropriate. Additionally, Dunette’s multiple comparisons test was performed. Testing was carried out with GraphPad Prism 9.1 (San Diego, CA, USA) software.

#### Microcomputed Tomography (μCT)

Mineral density analysis was performed using a μCT scanner (SkyScan 1174, Aartselaar, Belgium) with the following parameters: voltage: 52 kV, beam current: 790 mA, resolution: 6.7 μm, rotation: 360° in a 0.4° step, 0.25 mm aluminum filter. Calibration was performed using two phantoms (0.25 and 0.75 gHAp/cm^3^). Images were reconstructed using NRecon software (Version 1.6.9.8, SkyScan, Aartselaar, Belgium). Each specimen had two regions of interest (ROIs) outlined: (1) the lesion area and (2) an area of healthy enamel under it. The bone mineral density (BMD) was calculated using CTAn (1.14.4.1; Bruker micro-CT, Aartselaar, Belgium), and reported as % recovery:

(3) $$\%R=\frac{\left(∆Zd-∆Zr\right)}{∆Zd\times 100}$$

where ΔZd represents the difference between the area profiles of sound enamel and demineralized enamel, while ΔZr indicates the difference between the area profiles of sound enamel and remineralized enamel. Statistical testing for the μCT included Chi-Square goodness of fit test and multiple comparisons (with Bonferroni’s correction) (GraphPad Prism 9.1 (San Diego, CA, USA).

### 2.7. Remineralization of Artificial NCCLs and Their Analysis

After etching the artificial NCCL for 20 s, control specimens were incubated in artificial saliva at 37 °C for 7 days. P26-CS-treated samples were incubated with the hydrogel for 30 min following etching and then incubated in saliva. Reapplications were performed on days 3 and 5. NCCL surface morphology was analyzed using scanning electron microscopy (SEM) as described below:

#### Scanning Electron Microscopy (SEM)

NCCLs surface morphology was analyzed using scanning electron microscopy (SEM) (Nova NanoSEM 450, Thermo-Fisher, Waltham, MA, USA). Energy-dispersive X-ray spectroscopy (EDS) was performed using a detector (Thermo Fisher Scientific, Waltham, MA, USA) coupled to the SEM microscope. Prior to analysis, the samples were dehydrated and fixed with hexamethyldisilazane (HMDS) [12]. Samples were sputter coated with Pt/Pd for 45 s and analyzed under 10 kV.

## 3. Results

### 3.1. Degradation of P26 Peptide

In order to assess the self-degradation of the peptide P26 without enzymes, we incubated it for 7 days at 37°C. P26 produced small degradation products as determined by LC/MS (Table S1). Cleavages occurred at these defined amino acid residues: after the N-terminal methionine (M), before C-terminal D (aspartic acid) and/or before EVD, located near the C-terminus. Additionally, a small degree of dephosphorylation of Ser16 was paired with a loss of N-terminal residues (i.e., M, MPL, MPLP). In short, the P26 peptide was substantially resistant to self-degradation, which in turn indicates a prolonged shelf life. As expected, only a few terminal amino acid residues were naturally cleaved after 7 days, which suggested minimal loss of function in promoting mineralization.

### 3.2. Chitosan Diminishes Degradation of P26 by Salivary Enzymes

**Figure 1:**
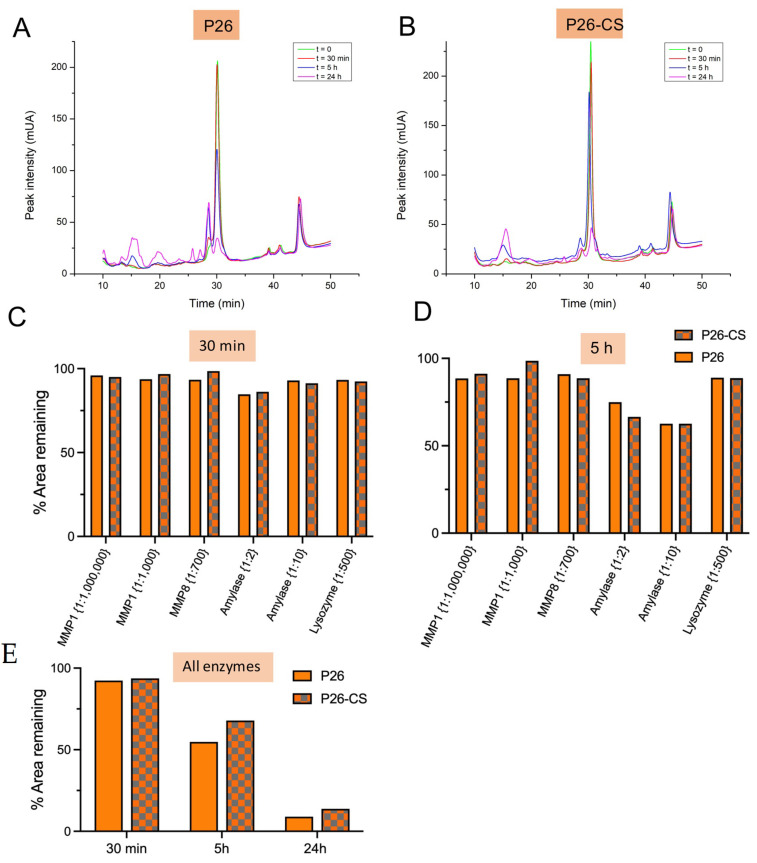
Peptide stability after incubation in artificial saliva with salivary enzymes. (**A**,**B**) HPLC spectra of P26 and P26-CS after incubation in artificial saliva with MMP1, MMP8, α-amylase, and lysozyme. Peptide stability was measured as the change in area of peptide peak (eluting at 30 min). Incubations for each sample were performed in triplicates (*n* = 3). (**C**,**D**) Peptide stability (as % of initial peptide peak area) of P26 and P26-CS (0.2 mg/mL) after incubation with individual enzymes for 30 min (**C**) and 5 h (**D**) at 37 °C. Enzymes were used at their native concentrations in humans. Ratios indicate amount of enzyme relative to peptide. (**E**) Peptide stability after incubation with all enzymes (native concentrations) at 37 °C (*n* = 3 per group).

Natural saliva contains numerous enzymes like amylase, lysozyme, and matrix metalloproteinases that may contribute to P26-CS degradation [30]. Peptide stability in the presence and absence of salivary enzymes was measured as the change in HPLC area of the peptide peak (eluting at 30 min) (Figure 1A,B) and computed as the % area remaining of the initial peak at t = 0 (Figure 1C–E). After a 30 min incubation with individual enzymes, P26 exhibited comparable stability levels regardless of the presence or absence of CS (Figure S1A–L). P26 in solution and in the CS hydrogel were both stable against enzymatic degradation with 90–100% of peptide substrate remaining after 30 min of reaction (Figure 1C). After 5h, peptide response to enzymatic activity varied (Figure 1D); for example, amylase promoted the highest levels of degradation amongst all the enzymes used. To test if this effect was due to the high concentration of amylase (enzyme to substrate ratio of 1:2), we tested a 5× dilution of amylase (now enzyme to substrate ratio 1:10). As expected, the HPLC spectra showed lesser degradation products (i.e., additional peaks other than the peptide peak) at the 1:10 ratio (Figure S1B,E) when compared to the native ratio of 1:2 (Figure S1A,D). In contrast, enzymes such as lysozyme and MMP8, whose naturally occurring levels are markedly low [30] had a mild degradation effect on the P26 peptide. P26 retention ranged from 88 to 98% for both enzyme groups (Figure 1D). Lastly, P26 was slightly more stable against MMP1 at both its native concentration (enzyme to substrate ratio of 1:1,000,000) as well as at 1000× concentration (1:1000 to peptide) in the presence of CS when compared to P26 only. Incubation with all the enzymes simultaneously at native concentrations showed that, while P26 and P26 with CS were comparably stable after 30 min, P26 with CS was markedly more stable after 5 h and 24 h (*p* > 0.05; Two-Way ANOVA) (Figure 1E).

### 3.3. Biocompatibility Evaluation of P26-CS

P26-CS hydrogel was defined as non-irritant, mild reactivity, and no evidence of sensitization, as per ISO standards (Table 1).

### 3.4. Presence of Salivary Enzymes Does Not Affect Enamel Remineralization

Using an in situ model of demineralized human teeth sections, we examined the remineralization efficiency of P26-CS in the presence of salivary enzymes. P26-CS has significantly enhanced enamel remineralization in demineralized sections [13].

**Figure 2:**
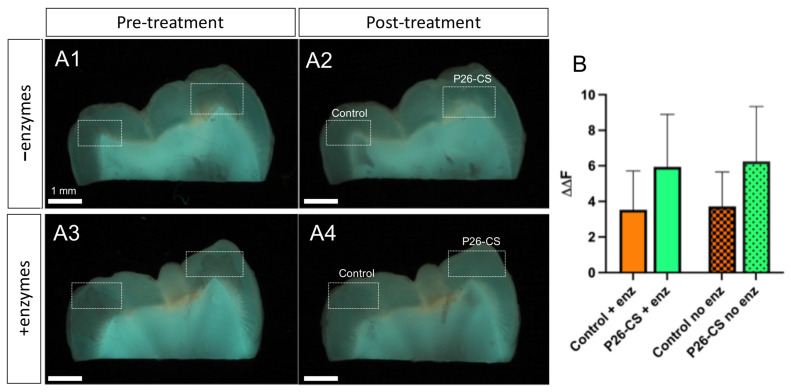
Evaluation of mineral density of demineralized enamel by QLF analysis after remineralization in the presence and absence of salivary enzymes. (**A1**–**A4**) Representative QLF images used to calculate ΔΔF_remin_, before treatment (**A1**,**A3**) and after treatment with P26-CS (**A2**,**A4**). Remineralization was carried out either in the presence (**A3**,**A4**) or absence (**A1**,**A2**) of salivary enzymes. (**B**) Statistical analysis of ΔΔF_remin_ values of samples remineralized with P26-CS with and without enzymes. No difference was observed between the ΔΔF_remin_ in groups with enzymes vs. the non-enzyme groups (dotted) (*p* > 0.05; One-Way ANOVA; *n* = 7/group). Additionally, treatment with P26-CS resulted in mineral density gain compared to controls both in the presence and absence of enzymes (*p* > 0.05).

Remineralization of enamel sections was assessed by calculating the change in mineral density (ΔΔF) by QLF analysis. The ΔΔF_remin_ was computed for each enamel window (Figure 2(A1–A4)). The results indicated that there was no difference in the ΔΔF_remin_ between the groups treated with and without enzymes (*p* > 0.05 by One-Way ANOVA; Figure 2B). This suggests that the presence of enzymes did not affect remineralization efficacy of P26-CS. Additionally, treatment with P26-CS resulted in mineral density gain compared to controls both in the presence and absence of enzymes (*p* > 0.05).

### 3.5. P26 Peptide Successfully Penetrated WSLs and NCCLs During Remineralization

#### 3.5.1. Peptide Penetration in WSL In Situ

After a 30 min treatment and remineralization with Cy5-P26-CS, peptide fluorescence was detected throughout the lesion (Figure S2A–C) spanning 749 μm along the z axis (Figure S2A). After remineralization in AS for 7 days at 37 °C, fluorescence intensity at the surface decreased by 83% (Figure S2D). Interestingly, the peptide could be detected through a greater depth within the lesion (873 μm) at t= 7 days than it was at t = 30 min. These findings indicate that the peptide continued to penetrate into deeper layers of the lesion after initial application. Treatment of the WSL with CS only yielded weak fluorescence (Figure S2E) that coincided with enamel autofluorescence (Figure S2F). Fluorescence was not detected in negative controls with etching only or healthy enamel (Figure S2G,J). By means of a fluorescently labeled P26 (i.e., Cy5-P26), we showed that a one-time etching application was sufficient to allow penetration of the peptide deep into the WSL.

**Figure 3:**
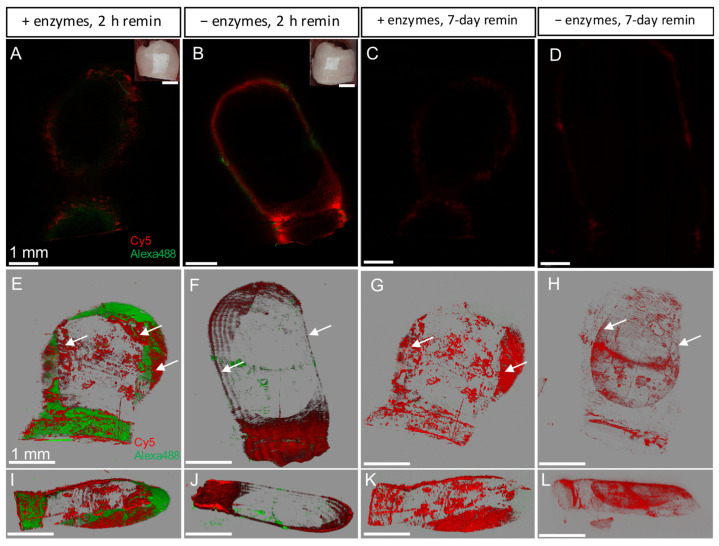
Penetration of Cy5-P26-CS on WSLs. After treatment, the specimens were remineralized in artificial saliva for 2 h with and without salivary enzymes (**A**,**B**, respectively). Insets in (**A**,**B**) show the white-light pictures of the block with the WSLs. Fluorescence was detected through a depth of ~390 μm. Fluorescence after 7-day remineralization at 37 °C from A (**C**) and B (**D**), respectively. Three-dimensional overlay front view (**E**–**H**) and lateral (**I**–**L**) from the z-stack showing peptide penetration (red) and enamel autofluorescence (green) from corresponding (**A**–**D**). White arrows indicate areas of peptide penetration.

To mimic the natural oral environment where salivary enzymes are present, the remineralization was conducted in the presence of salivary enzymes. P26-CS hydrogel application was followed by a 2 h remineralization in artificial saliva at 37° C. A decrease in fluorescence intensity was observed compared to the control, likely because of enzymatic processing (Figure 3A,B). However, examining comparable depth z-stacks indicated that the peptide penetration depth was similar both the test and control samples, measuring between 380 and 390 μm (Figure 3A,B). Importantly and as seen in the 3D overlays, enough peptide remained in the WSL after a 7-day remineralization, both in the presence (Figure 3C,G,K) and absence of enzymes (Figure 3D,H,L).

#### 3.5.2. Peptide Penetration in Artificial In Situ NCCLs

**Figure 4:**
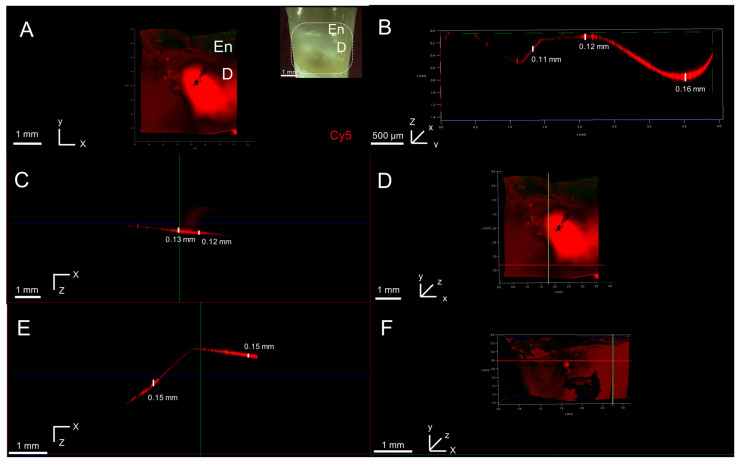
Cy5-P26 treated in situ NCCL. (**A**) Two-dimensional sectioning of in situ lesion after 2 h remineralization. Inset: white-light picture of the lesion. (**B**) Three-dimensional volume rendering of lateral view of lesion showing peptide penetration along the YZ plane. (**C**–**F**) Representative images of peptide penetration along the XZ plane after 1.5 h (**C**) and 7 days remin. (**E**) along with the point where it was measured in the lesion (**D**,**F**), respectively. En: enamel; D: dentin.

We successfully created in situ NCCLs at or below the CEJ level, with depths ranging from 0.4 to 0.9 mm (Figure S3). Using our artificial in situ NCCL model, we wanted to investigate if the peptide would penetrate in the affected cervical dentin. The NCCL was treated with Cy5-P26-CS and subsequently analyzed after a 1.5 h (Figure 4A–D) and a 7-day remineralization cycle (Figure 4E,F) using confocal microscopy. After 1.5 h of remineralization, the penetration alongside the lesion varied slightly, ranging from 0.11 mm to 0.16 mm (Figure 4B). In the XZ plane, the average penetration was 0.12 mm (Figure 4C,D) and after 7 days it increased to 0.15 mm (Figure 4E,F).

#### 3.5.3. Peptide Penetration in Natural NCCL

**Figure 5:**
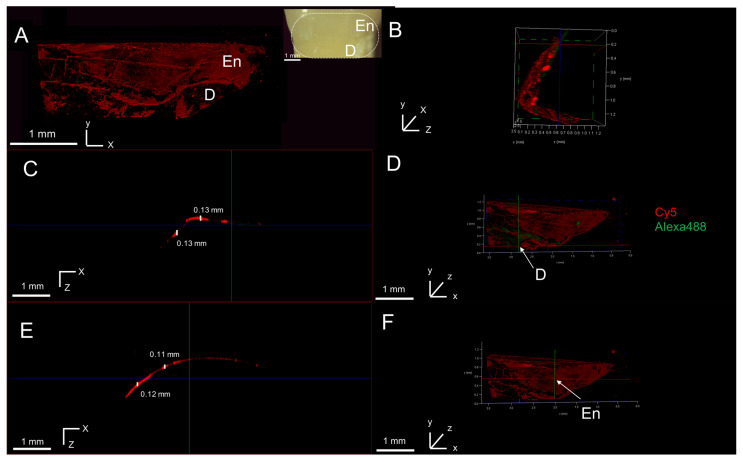
Cy5-P26 treated in natural NCCL. (**A**) Lesion after 1.5 h remineralization showing peptide presence in enamel and dentin. Inset: white-light picture of lesion. (**B**) Lateral view of lesion showing peptide penetration along YZ plane. (**C**–**F**) Representative images of peptide penetration along the XZ plane in dentin (**C**) and enamel (**E**) along with the point where it was measured in the lesion (**D**,**F**), respectively. En: enamel; D: dentin.

The top half of the natural NCCL containing affected enamel and dentin was etched and then treated with the hydrogel. The analysis showed that the peptide was present in both tissues and had adhered primarily to the boundary between enamel and dentin (Figure 5A,B). Penetration depth was found to be similar in both tissues, with the average penetration in dentin was 0.13 mm (Figure 5C,D) and in enamel 0.12 mm (Figure 5E,F). These results support the notion that the hydrogel could serve as treatment for both tissues, which can be simultaneously affected in NCCLs.

### 3.6. P26-CS Treatment Improved Mineral Density in WSLs

**Figure 6:**
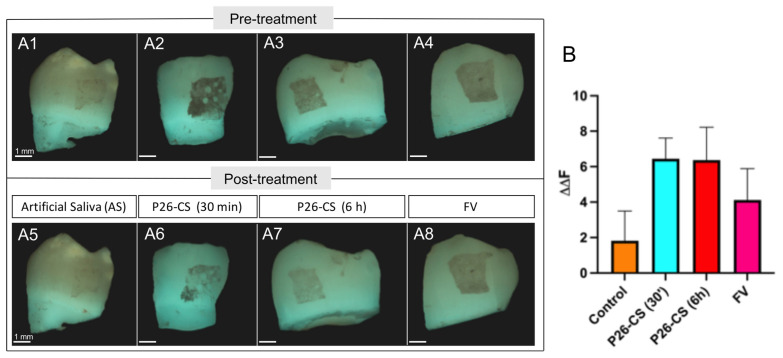
Assessment of mineral density of WSLs after a 21-day remineralization cycle. (**A1–A8**) Representative QLF images used to calculate ΔΔF_remin_ showing the fluorescence of the lesion pre- and post-treatment. (**B**) Quantitative analysis of the QLF images. Treatment with P26-CS resulted in the highest ΔΔF_remin_. Application of P26-CS for 30 min, 2× per week (blue) or 6 h, 1× a week (red) had an almost identical effect in the ΔΔF_remin_. *p >* 0.05 (*p* = 0.17 by One-Way ANOVA; *n* = 8 per group. Error bars represent standard error of the mean). FV = Fluoride Varnish (positive control).

After showing successful penetration of the P26 peptide into the WSL, we wanted to determine how P26-CS remineralized WSLs with the inclusion of an etching step. WSLs showed the highest average gain in fluorescence in QLF after 21-day remineralization in AS when treated with P26-CS (0.5 mg/mL), and when compared to fluoride varnish or artificial saliva (control) (Figure 6). Moreover, the length and frequency of P26-CS hydrogel application did not influence its observed remineralizing potential. Both P26-CS groups, whether treated for 30 min twice a week or 6 h once a week during the 21-remineralization period, showed almost identical ΔΔF_remin_ (Figure 6B).

Based on the observation that there was no difference between 21 days and 30 min, we selected the 30 min, 2× a week protocol for subsequent experiments with the WSLs model. Shorter, more frequent applications can facilitate a smoother experience when it comes to patient care.

**Figure 7:**
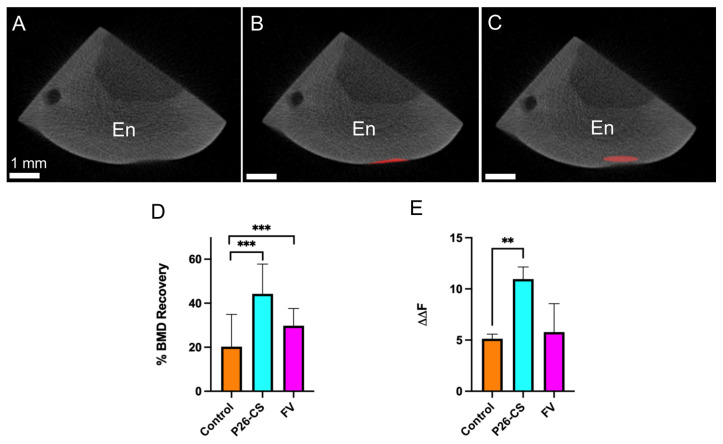
Assessment of mineral density and bone mineral density. (**A**–**C**) Representative microcomputed tomography cross section images of a block after remineralization and treatment with P26-CS (30 min, 2× a week). The ROI function (in red) was used to circumscribe the area of interest and calculate the bone mineral density (BMD) of the lesion area (**B**) as well as healthy enamel (En) above the lesion (**C**). (**D**) BMD recovery (as %) of the demineralized enamel after 21-day remineralization (*p* < 0.0001, Chi-Square test, *n* = 4/ group) Multiple comparisons test, after Bonferroni’s correction, showed a significant difference between control and P26-CS, and control and FV (*** *p* < 0.001). (**E**) Quantitative analysis by QLF showed a significant difference in ΔΔF_remin_ amongst the groups (*p* = 0.015, Brown–Forsythe and Welch ANOVA, *n* = 4/ group), and by pairwise comparison, a significant difference between the mean ΔΔF_remin_ of control and P26-CS (Dunette’s pairwise comparison, ** *p* = 0.002) was observed.

The gain in mineral density suggested by the QLF analysis was further tested by assessment of the change in bone mineral density (BMD) of the lesion area. The specimens were simultaneously analyzed using QLF and using microcomputed tomography (μCT) (Figure 7A–C). Analysis were performed after demineralization and once again after a 21-day remineralization in AS. The data showed that the treatment was statistically significantly associated with the recovery of bone mineral density (*p* < 0.0001) (by chi-square test) (Figure 7D), with P26-CS group presenting the highest % recovery, about twice of that observed in the control group. Furthermore, the ΔΔF_remin_ was statistically significantly different in control and P26-CS groups (Figure 7E). P26-CS also performed better than FV, providing an improvement of 1.5× in % BMD recovery and ΔΔF (Figure 7D,E). Taken together, these results further supports that the P26-CS hydrogel successfully mediates recovery of mineral density in artificial WSLs in situ.

### 3.7. P26-CS Treatment of NCCL Lesions Promoted Crystal Growth in Dentin and Enamel

**Figure 8:**
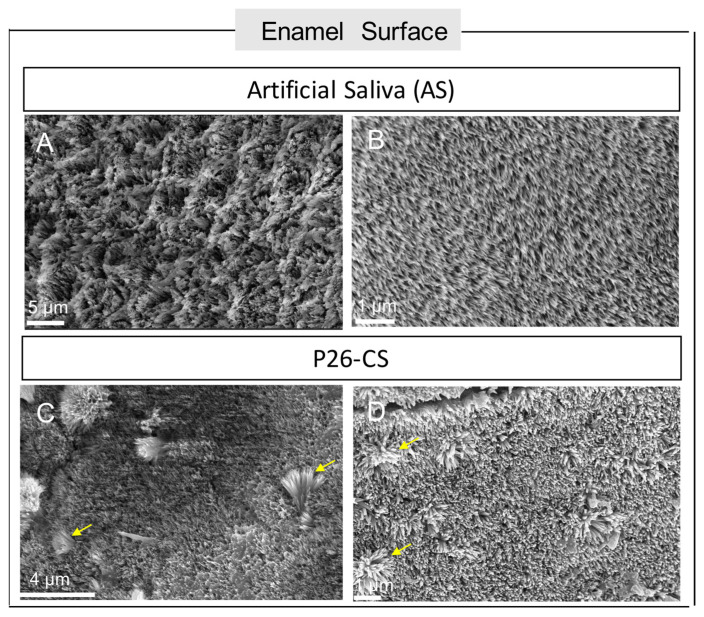
Surface morphology of enamel. (**A**,**B**) Enamel of NCCLs after etching and subsequent remineralization in artificial saliva for 7 days displayed tiny crystals. (**C**,**D**). Enamel of NCCLs after etching and P26-CS treatment showed bigger crystals, with bundles of longer crystals (arrows) scattered around the surface.

After 7-day remineralization, SEM was used to observe the morphology of enamel and dentin surfaces in NCCLs. In artificial saliva, remineralized enamel was characterized by tiny, loose crystals (Figure 8A,B). In contrast, P26-CS-treated enamel presented tightly packed, rod-like crystals where clusters of longer crystals were often identifiable (Figure 8C,D).

**Figure 9:**
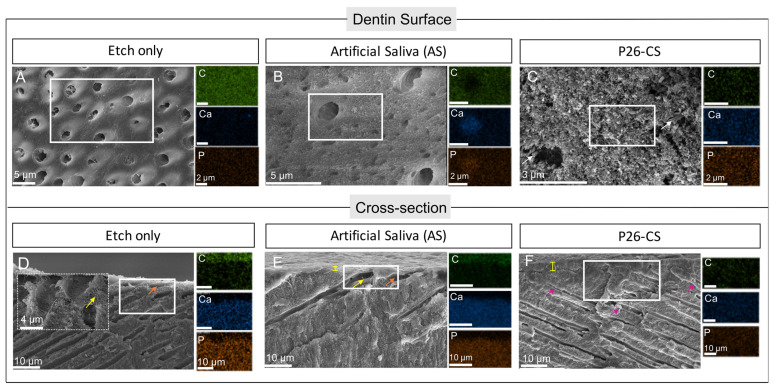
Dentin morphology along the surface and cross section orientations. (**A**–**C**) Dentin surface after etching (**A**), remineralization in AS (**B**), and P26-CS treatment (**C**) with corresponding elemental analysis of carbon (C), Calcium (Ca), and phosphorous (P). Etching resulted in an almost complete removal of Ca and P minerals from the surface (**A**). Remineralization in AS deposited a few minerals on the surface (**B**). Mineral content was the highest in the P26-CS-treated sample **(C)**, which also exhibited a layer of crystals that covered the tubular orifices. White arrows in (**C**) point to needle-shaped crystals. (**D**–**F**) Dentin morphology along the cross-sectional orientation. (**D**) Etch only was characterized by an absence of a superficial layer, empty tubules (orange arrow) and exposed collagen fibrils (inset, yellow arrow). (**E**) AS group presented a mostly organic superficial layer, which was narrow and characterized by exposed collagen fibrils (yellow arrow) and empty tubules (orange arrow). (**F**) After P26-CS treatment, a thicker superficial layer with markedly decreased organic content was observed, in addition to abundant deposits within the tubules (pink arrows). Insets (white boxes) show area where the EDS maps were taken.

After etching, the dentin surface was void of inorganic minerals, namely calcium and phosphorous, as confirmed through EDS (Figure 9A). Remineralization using artificial saliva alone resulted in a minimal deposition of minerals on the surface (Figure 9B). Importantly, the remineralization of dentin with P26-CS resulted in needle-shaped crystals of considerable length that were tightly packed, thereby completely occluding the dentinal tubules (Figure 9C).

Cross-sectional data further confirmed these findings. As expected, the etch-only group showed a complete demineralization on the surface, with collagen fibrils clearly visible (Figure 9D). Similarly, the AS group showed exposed collagen fibrils in conjunction with a thin, highly organic superficial layer (Figure 9E). In contrast, P26-CS-treated specimens presented a thicker layer of ~3.5 μm above the native dentin, as well as a notable amount of mineral deposits within the tubules (Figure 9F). The new dentinal layer was tightly bound to the underlying dentin and could withstand 5 min of ultrasonication. These results indicate that the hydrogel promoted remineralization in three ways: (1) mineral deposition within the tubules, (2) deposition of a new layer, and (3) crystal growth in dentin.

## 4. Discussion

Early treatment of incipient caries lesions such as WSLs is an essential step in improving the prognosis of teeth with dental caries and mitigating the need for invasive dental interventions [33]. For many years, the preferred method for the prevention and treatment of early carious lesions and WSLs has been fluoride varnishes [34]. However, the inability of the fluoride ions from the varnish to penetrate deeper into the lesion poses a major limitation to their usage. Thus, the actual lesion which lies in the subsurface layer is not repaired and only a hardened surface layer is formed [35,36]. Additionally, excessive levels of fluoride exposure have been linked to neurotoxicity and mitochondrial damage [37,38,39]. In contrast, emerging alternatives that use bioactive peptides which are non-toxic and biocompatible, like the P26-CS hydrogel, offer additional benefits while actively repairing damaged dental tissues. We demonstrated here that P26-CS is a biocompatible biomimetic tool, as it has the potential to regenerate rather than just repair. With the implementation of a simple etching step, we achieved penetration of the P26 peptide into the deeper layers of WSLs (~300 μm), a significant improvement when compared to prior efforts [13]. Moreover, treatment with P26-CS doubled mineral density recovery (compared to control) and surpassed that of the fluoride varnish group by a 1.5× gain.

The mechanism of remineralization by P26-CS is likely governed by P26-mediated stabilization of Ca-P clusters. This facilitates the organization of Ca-P clusters into linear chains, which subsequently develop into enamel-like, co-aligned crystals integrated with the natural enamel substrate [29]. The crystals subsequently develop on the enamel in a well-organized fashion, aligning their axes perpendicularly to the enamel surface, guided by the sustained regulation of P26. In addition to promoting remineralization, the pH responsiveness of P26-CS can offer protection against erosion. This is due to the protective layer formed by CS capable of sequestering acidic hydrogen ions, thus protecting the enamel during acidic challenges.

NCCLs pose unique challenges for restoration due to the varying morphologies and surface characteristics that they present with [1]. NCCLs are characterized by a non-carious loss of cervical structure and occur in a wide range of morphologies (e.g., wedge-shaped, notched, etc.) [40]. The variability in these lesions is further increased by the extent of tissue lost during the process. For example, in some cases enamel is visibly affected, whereas in others enamel is minimally compromised, as the lesion may be located at or below the cementoenamel junction [41]. Additionally, pathological and physiological changes have been reported in dentin (e.g., hypermineralization, sclerotic dentin, etc.) [9,42,43]. Such changes have been suggested to markedly decrease bonding in self-etch and total-etch systems [42] and ultimately cause failure of resin restorations due to a lack of micromechanical retention [9]. Overall, the significant variability renders the clinical management of NCCLs to be especially difficult and further presents a hurdle in replicating them in laboratory environments.

To date, only a limited number of studies have successfully simulated NCCLs in vitro [10,44]. In the present study, we successfully simulated NCCLs in situ using healthy human premolars. The in situ lesions exhibited varying shapes with a loss of tissue in the cervical region of the tooth, ranging from 0.5 mm to 1.5 mm in depth. This variability provided a comprehensive model that recapitulates the different clinical presentations of NCCLs. Additionally, the surface morphology of the in situ lesions exhibited many features of clinical NCCLs such as furrows, cracks, dimples, and a mixture of open and closed tubules [45].

Here, we used different models to (1) characterize P26 peptide degradation in vitro, (2) examine biocompatibility of P26, (3) evaluate the effect of salivary enzymes on (a) P26-CS degradation in vitro and (b) enamel remineralization after P26-CS treatment in vitro, and (c) study the efficacy of P26-CS in repairing in situ WSLs and NCCLs, including an assessment of peptide penetration into the lesions.

As documented by mass spectrometry data, we demonstrated that even after some self-degradation of the P26 peptide, enough bioactive domains remained which in turn can function to promote remineralization on enamel sections. When evaluating the effects of salivary enzymes on P26-CS degradation, we showed that CS conferred additional protection against degradation to the P26 peptide. When amylase was used in an enzyme/substrate ratio of 1:2, which is the native ratio that is seen in humans, the degradation was greater than that of the diluted 1:10. While these results were expected, it is important to note that the native enzyme ratios used did not affect remineralization by P26-CS in the enamel sections. Additionally, the presence of CS made MMP1 more stable than P26 alone. This example further exemplified the protective role of CS in the hydrogel without impacting its function. Also, CS did not impede peptide release from the hydrogel, as confirmed in the penetration and remineralization experiments. This finding points to a protective effect of CS, which would grant extended shelf-life stability of the product. Additionally, our data show that the presence of salivary enzymes did not impede the remineralization potential of P26-CS when tested on demineralized enamel sections. Thus, P26-CS-treated samples exhibited an almost identical gain in mineral density (ΔΔF ± 1%) with and without salivary enzymes. Using a fluorescently labeled peptide (Cy5-P26), we showed that P26-CS successfully penetrated WSLs in vitro, both in the presence and absence of salivary enzymes, and further remained detectable in the lesion for 7 days. Our QLF and BMD results concomitantly showed that P26-CS promoted a superior gain in mineral density when compared against control and FV groups (*p* < 0.05). While the remineralization of WSLs was confirmed by a significant gain in mineral density, the lesions did not show any visible optical changes. Lastly, using a novel model of in situ NCCLs, we showed how P26-CS mediated repair in both enamel and dentin tissues. P26-CS-directed biomimetic crystal growth and assembly in dentin resulted in the generation of a layer of needle-shaped crystals that were observed covering the dentinal tubules. Similarly, P26-CS facilitated growth of longer and more tightly packed crystals compared to those seen in the control etched enamel of the NCCLs remineralized using AS alone.

In this study, an etching step was incorporated prior to P26-CS application which maximized peptide penetration into the in situ lesions. This improved penetration resulted in a greater depth of remineralization. Etching also resulted in more effective adhesion of the hydrogel to the enamel surface by potentially maximizing the electrostatic interaction between enamel and the positively charged chitosan [26]. Enhanced adhesion is beneficial as it can promote the recruitment of inorganic ions (Ca^2+^, PO_4_^3−^) by charged residues of the peptide [11], which can then be subsequently guided into the subsurface location of the lesion. Surface etching is commonly employed in clinical dentistry and is regarded as a minimally invasive practice that can improve treatment success [35]. In light of the results of this study, incorporation of an etching step with a mild acid (e.g., phosphoric acid) prior to the first application of P26-CS will be necessary to achieve the desired results when used in future clinical applications.

## 5. Conclusions

We used human molars and pre-molars as in situ models to investigate the efficacy of a biocompatible amelogenin peptide chitosan (P26-CS) in repairing artificial dental lesions such as WSLs and NCCLs. The stability experiments suggest that incorporating our peptide, P26, into a CS-based hydrogel will increase its shelf life. Cytotoxicity reports validated ISO compliance of the P26-CS hydrogel. P26-CS was effective in remineralizing enamel in the in situ model of subsurface lesions. In the NCCL model, P26-CS treatment had a dual effect by promoting the remineralization of both enamel and dentin. Further, our results demonstrate that P26-CS not only enhanced mineralization in etched enamel sections but also improved the surface morphology of both enamel and dentin, suggesting its potential as a therapeutic agent in clinical applications for WSLs and NCCLs. To evaluate the clinical potential of P26-CS, we will consider future experiments that will advance the application of P26-CS in human clinical trials. Some of these experiments could explore the efficacy of remineralization using human saliva as well as the evaluation of mechanical strength and bonding of the new layer to the underlaying tooth substrate.

## Figures and Tables

**Table 1:** Cytotoxicity, sensitization, and oral mucosa irritation results from independent testing.

	Objective	Methods	Key Results
**Dermal Sensitization**	Assess potential for dermal sensitization	Guinea pig maximization test (GPMT) and Buehler test	No evidence of sensitization observed in test subjects compared to controls
**Oral Mucosa Irritation**	Evaluate effects on oral mucosa	Oral mucosal irritation test on rabbits	Minimal irritation observed, classified as non-irritant
**Cytotoxicity**	Determine cytotoxic potential of materials	In vitro assay using L929 mouse fibroblast cells	Moderate reduction in cell viability, within acceptable limits for cytotoxicity tests

## Data Availability

Data may be provided upon request from the corresponding author.

## Supplementary Materials

The following supporting information can be downloaded at: https://www.mdpi.com/article/10.3390/biomimetics10020120/s1, Table S1: Degradation products of P26 aqueous solution after 7 days at 37 °C observed by LC/MS analysis; Figure S1: HPLC spectra of P26 and P26-CS after incubation in artificial saliva with MMP1, MMP8, a-amylase, and lysozyme; Figure S2: Treatment of WSLs in situ visualized with confocal microscopy; Figure S3: In situ NCCLs on human premolars.
