# Supplementary material for: Treating White Spot Lesions and Non-Carious Cervical Lesions with Amelogenin Peptide-Based Hydrogel
Source: Biomimetics (Basel). 2025 Feb 18;10(2):120. doi: 10.3390/biomimetics10020120 (PMC11853660; doi:10.3390/biomimetics10020120)
Supplement: Supplementary file 1 [file biomimetics-10-00120-s001.zip › biomimetics-3457062-supplementary.pdf]

Supplementary material

Treating White Spot Lesions and Non-Carious Cervical Lesions with Amelogenin Peptide-Based Hydrogel

Erika Bauza Nowotny [1], Salony Jassar [1], Jin-Ho Phark [2] and Janet Moradian-Oldak [1,3,*]

[1] Center for Craniofacial Molecular Biology, Herman Ostrow School of Dentistry, University of Southern California, Los Angeles, CA, 90089 USA; nowotny@usc.edu (E.B.N.); sjassar@usc.edu (S.J.)
[2] Department of Operative Dentistry, Herman Ostrow School of Dentistry, University of Southern California, Los Angeles, CA, 90089USA; phark@usc.edu (J.-H.P.)
[3] Department of Biomedical Engineering, Viterbi School of Engineering, University of Southern California, Los Angeles, CA, 90089 USA
* Correspondence: joldak@usc.edu (J.M.-O.)

Table S1: Amino acid sequence and degradation products of P26 in aqueous solution after 7 days at 37°C, determined by LC/MS analysis. In **bold**: Amino acid residues cleaved.

P26	**Degradation**	**Sequence**	**Peak (m/z)**
	M	**M**PLPS(PHOS)YEVLTPLKWPSTDKTKREEVD	430.47
	M dephosphorylation	**M**PLPS**(PHOS)**YEVLTPLKWPSTDKTKREEVD	732.36
	MPL dephosphorylation	**MPL**PS**(PHOS)**YEVLTPLKWPSTDKTKREEVD	680.36
	EVD	MPLPS(PHOS)YEVLTPLKWPSTDKTKRE**EVD**	564.29
	D	MPLPS(PHOS)YEVLTPLKWPSTDKTKREEV**D**	756.88

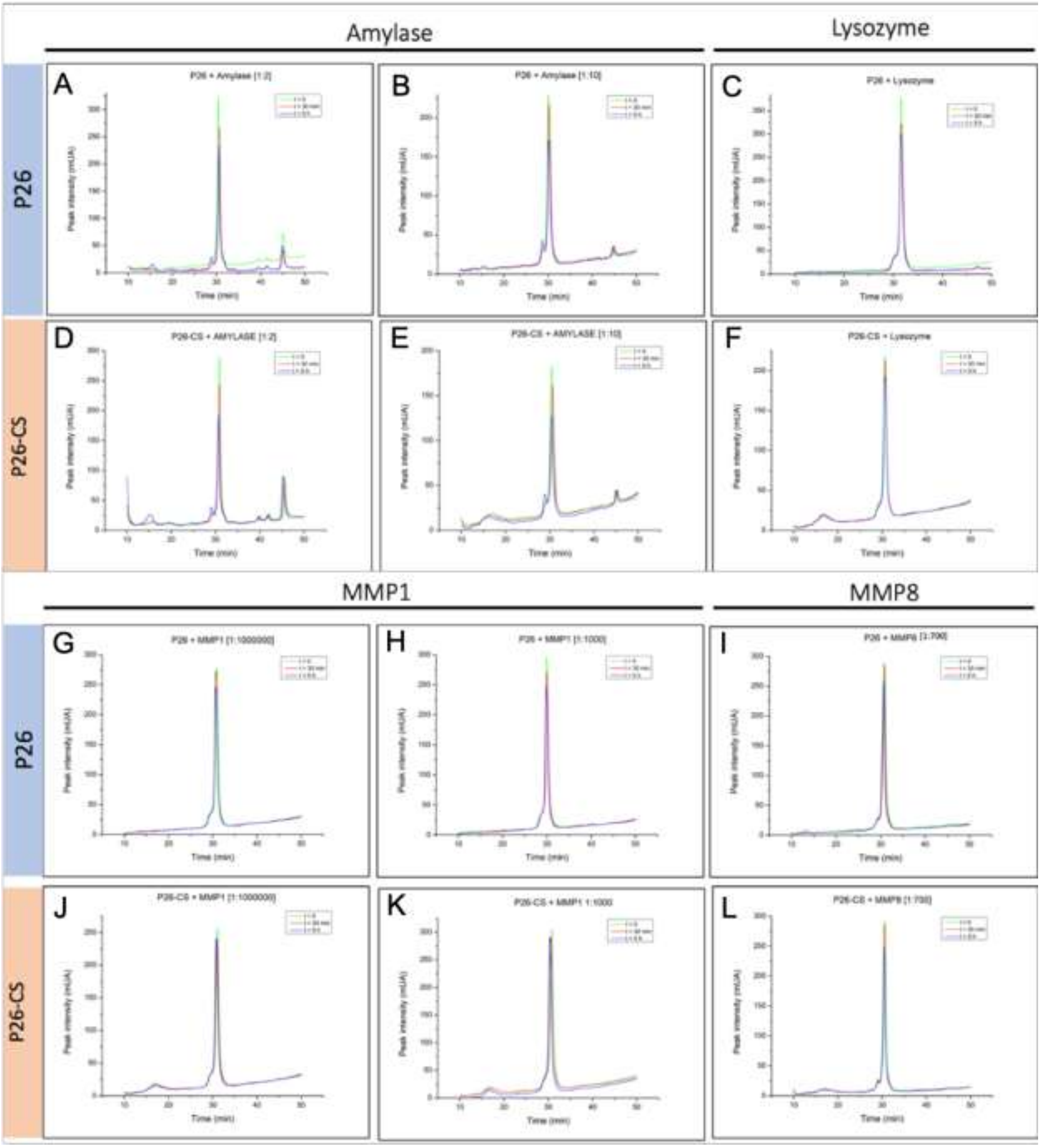

Figure S1. (A-L) HPLC spectra of peptide alone (P26) and incorporated in CS (P26-CS) after incubation in artificial saliva with MMP1, MMP8, a-amylase, and lysozyme. All enzymes were tested in concentrations found in humans. Amylase **(B,E)** and MMP1 **(H,K)** were tested in additional concentrations (1:10 and 1:1000, respectively).

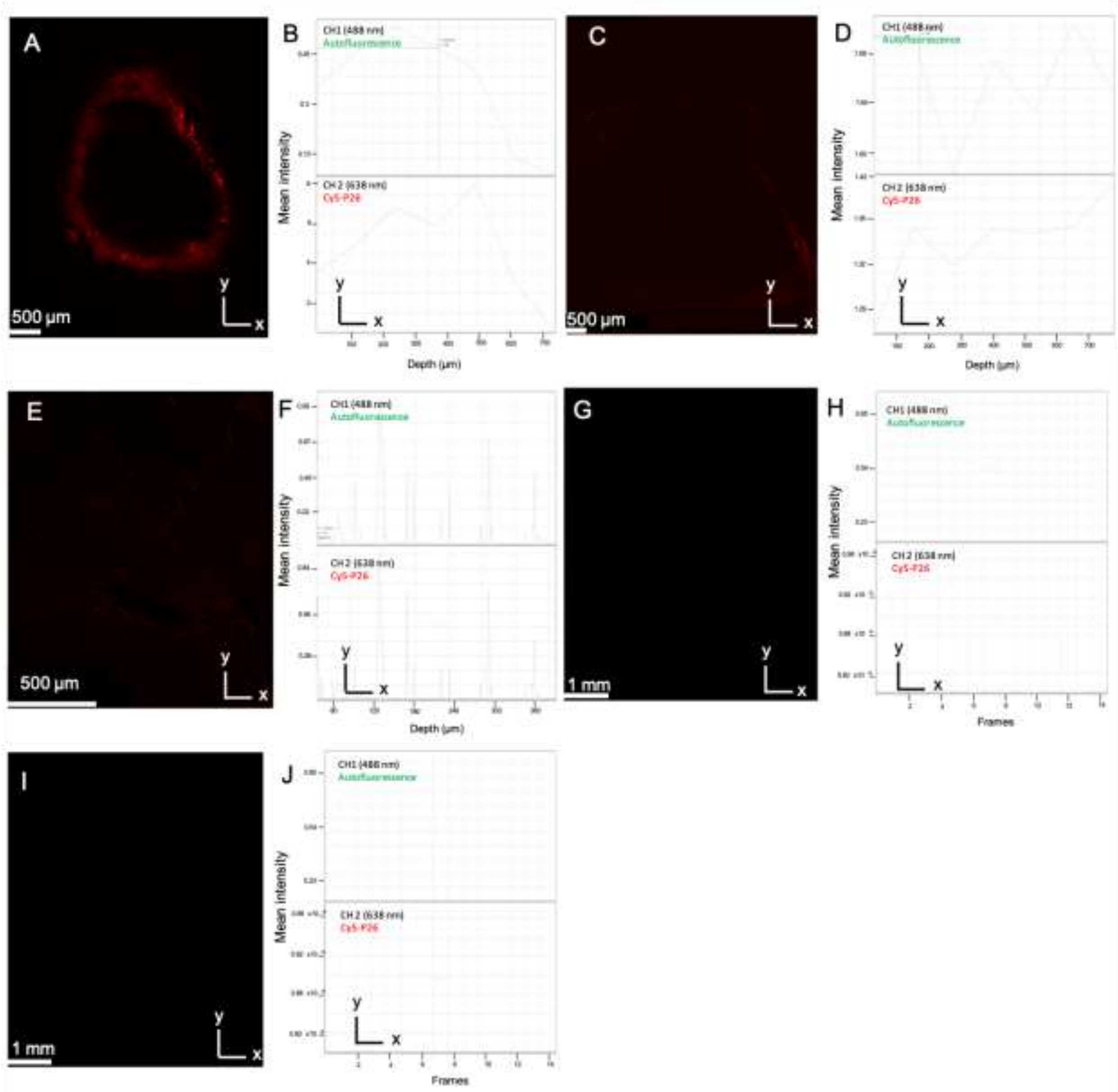

Figure S2. Treatment of WSLs in situ visualized with confocal microscopy. **(A)** Representative Z-stack image showing Cy5-P26-CS on the lesion after a 30 min treatment application. (**B**) Fluorescence intensity of Cy5-P26 was detected up to a volume depth of 749 µm, (**C**) A faint fluorescence was observed after Cy5-P26-CS treatment followed by 7-day remineralization in AS at 37°C. (**D**) Fluorescence intensity of Cy5-P26 showed that volume depth increased to 800 µm depth after 7 days remineralization. (**E,F**) Minimal fluorescence was detected after treatment with CS only. (**G-J)** No fluorescence was detected in etching only (G, H) or healthy enamel (I,J).

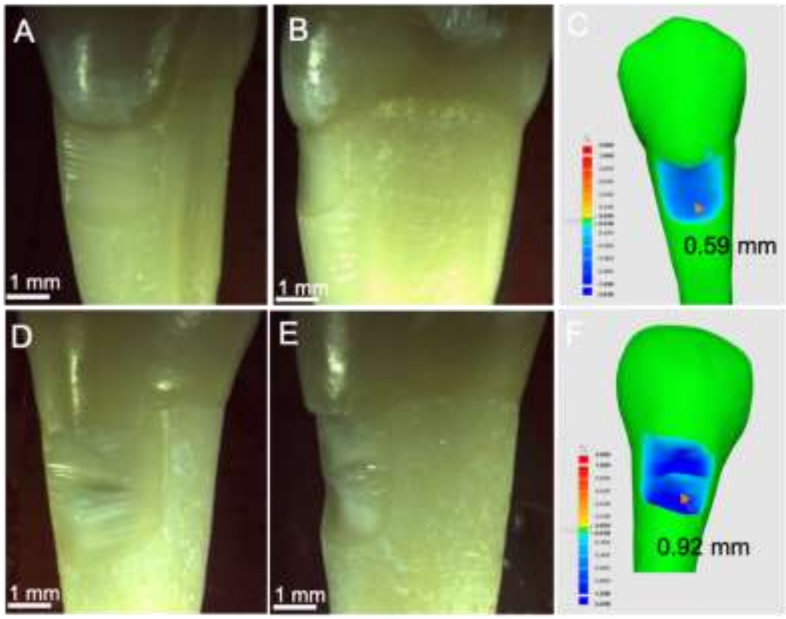

Figure S3. In situ NCCLs on human premolars. **(A, B, D, E)** White light pictures of front and side of lesion. **(C, F)** 3D representation showing the depth of the lesion.
